# A Performance Evaluation Scheme for Multiple Object Tracking with HFSWR

**DOI:** 10.3390/s19061393

**Published:** 2019-03-21

**Authors:** Kun Wang, Pengju Zhang, Jiong Niu, Weifeng Sun, Lun Zhao, Yonggang Ji

**Affiliations:** 1 College of Engineering, Ocean University of China, Qingdao 266100, China; kunwang@ouc.edu.cn (K.W.); kafchang@outlook.com (P.Z.); zhaolun@stu.ouc.edu.cn (L.Z.); 2 College of Information and Control Engineering, China University of Petroleum, Qingdao 266580, China; swf0217@163.com; 3 The First Institute of Oceanography, State Oceanic Administration, Qingdao 266061, China; jiyonggang@fio.org.cn

**Keywords:** multiple object tracking, HFSWR, performance evaluation, target motion analysis, motion pattern metrics, evolutionary computation

## Abstract

High-frequency surface wave radar (HFSWR) can detect and continuously track ship objects in real time and beyond the horizon. When ships navigate in a sea area, their motions in a time period form a scenario. The diversity and complexity of the motion scenarios make it difficult to accurately track ships, in which failures such as track fragmentation (TF) are frequently observed. However, it is still unclear how and to what degrees the motions of ships affect the tracking performance, especially which motion patterns can cause tracking failures. This paper addresses this problem and attempts to undertake a first step towards providing an intensive quantitative performance assessment and vulnerability detection scheme for ship-tracking algorithms by proposing an evolutionary and data-mining-based approach. Low-dimensional scenarios in terms of multiple maneuvering ship objects are generated using a grammar-based model. Closed-loop feedback is introduced using evolutionary computation to efficiently collect scenarios that cause more and more tracking performance loss, which provides diversified cases for analysing using data-mining technique to discover indicators of tracking vulnerability. Results on different tracking algorithms show that more cluster and convergence patterns and longer duration of our convoy and cluster patterns in the scenarios can cause severer TF to HFSWR ship tracking.

## 1. Introduction

High-frequency surface wave radar (HFSWR) has been investigated as a platform for detection and tracking of moving ships in surveillance systems due to its advantage of all-day operation with low cost and wide observable region. However, HFSWR systems exhibit many shortcomings that degrade the detection performance, such as poor range and azimuth resolution, high nonlinearity in the state/measurement space, and significant false alarm rate (FAR) due to both sea clutter and man-made/natural interference [1]. Different detection methods have been proposed to properly address these issues, such as various constant false alarm rate (CFAR) detection algorithms, curvilinear regression analysis, and adaptive detection technique [2]. Precise estimation of which azimuth ship object echo comes is one of the crucial problems. To improve the accuracy of ship objects’ direction of arrival (DOA) estimation, beam forming and direction finding techniques have been applied [3,4]. However, their performances are “easy to be affected by array uncertainties such as sensor position error and gain/phase errors” [4], which may introduce large errors into DOA estimations of up to 10${}^{\circ }$ [5]. For these reasons, ship targets’ kinematic (e.g., range, azimuth, and range rate/radial velocity) information detected using a HFSWR system can suffer from low accuracy.

Modern multitarget tracking (MTT) technique is mandatory to deal with the aforementioned shortcomings and to obtain operationally acceptable detection performance [1]. The objective of a HFSWR-based ship-tracking algorithm is to associate consecutive motion states of ship targets into corresponding tracks. The last two decades have seen significant advances in ship tracking algorithms [6,7,8].

However, intensive and quantitative performance assessment to HFSWR-based ship-tracking algorithms has been a formidable challenge due to the presence of many vulnerabilities, such as man-made interference, ionospheric variation, high waves [6] and motions of ship targets [7,9]. To address the former signal-level vulnerability, Park et al. [6] proposed a simulator based on compact HF radar in 2017, in which ship signal, sea surface signal, random clutter and background noise is modeled to provide a guideline for effective assessment of ship detection and tracking algorithms.

In terms of the impact of motions of multiple ship targets on tracking for HFSWR, it is still unclear about how and to what degrees the impact is, especially about which types of “scenarios” or “motion patterns” can cause performance loss or failures to a HFSWR ship-tracking algorithm. A **scenario** is a description of what happens on the sea, specifically a description of the motion states of the ships navigating in a certain sea area within a time period. It has been accepted that maneuvering or multiple objects may cause tracking failures such as false targets, loss targets or track fragmentation (TF) [1,7,8,10]. Recent work has revealed that tracking performance may suffer seriously due to the residual effects of disappearing ships or the interference when ships are in close proximity [7].

Consequently, the phenomenon of track fragmentation (TF) is evident even in ship-tracking algorithms with good overall performance [1,10,11]. TF is a phenomenon in tracking when the track of a ship breaks into several subtracks. TF is mainly due to the lack of target contacts for some periods of time. Possible reasons for this problem include the radar synchronisation turning off and targets sailing in the clutter peaks associated with Bragg scatter [10,12]. We demonstrate in this paper that some motion patterns can also cause TF to HFSWR ship tracking.

However, understanding the effect of motions of multiple ship targets on tracking is not a trivial task because of the diversity and complexity of scenarios. For one thing, the number of ship targets in the scenario is uncertain, ranging from one to hundreds and thousands. For another, every ship target is possible to move autonomously to a great extent even under the convention on the international regulations for preventing collisions at Sea, 1972 (COLREGs).

Existing attempts rely on simulations or automatic identification system (AIS) as ground-truth data for evaluating the performance of a HFSWR ship-tracking algorithm. These works have helped us to understand the performance of a tracking algorithm while suffering from the following defects at this stage, which imposes **challenges on effective quantitative performance assessment and vulnerability detection of HFSWR ship-tracking algorithms**: **(1)** The test dataset fails to incorporate diversified scenarios that can reflect the autonomous movement of ships in the radar’s surveillance area in a given time period. For instance, the simulated data are generated from a specific motion model, such as the Markov model or straight line model [7,13], with few ship targets (mostly fewer than five or only one for maneuvering ship targets) and the initial states manually assigned. The AIS data for testing [8,10] only represents one or more cases obtained from a specific sea area in a specific time period. **(2)** The dimension of the testing data is high since the simulated or AIS data can reach up to tens of thousands of sampled states of ship targets. **(3)** It is challenging to obtain cases or datasets with varying degrees of performance characteristics, especially those scenarios that could cause severe tracking performance loss. We conjecture that Challenge (2), the high dimensionality of data, is partly to blame, thus it is difficult to collect interesting scenarios (i.e., testing cases) through searching.

**Figure 1:**
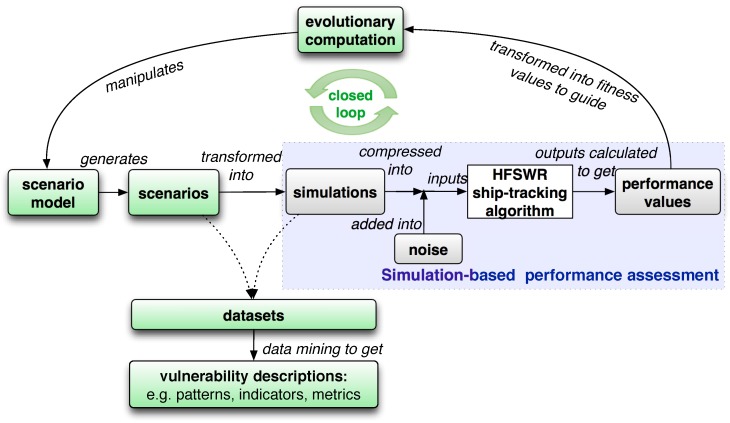
System block diagram: The blue block illustrates the existing simulation-based performance assessment method for the HFSWR ship-tracking algorithm, while the green blocks highlight the contributions of our approach, with the label on each of the directed lines denotes the relationship between or the operation involved in the two connected entities or concepts in the blocks.

To meet the above three challenges, we propose an **evolutionary and data-mining-based approach that attempts to undertake a first step towards providing an intensive quantitative performance evaluation and vulnerability detection scheme for HFSWR ship-tracking algorithms**. Figure 1 presents the system block diagram of our approach. The green blocks highlight the contributions of our approach compared with existing simulation-based performance evaluation method—which is similar to the AIS-based method, just by transforming the AIS data into the “inputs” of the HFSWR ship-tracking algorithm—illustrated in the blue block.

To address Challenges (1) and (2), a grammar-based scenario model that can capture the motions of ships on the semantic level is defined and derived to generate various cases for testing the performance of a HFSWR ship-tracking algorithm (see “scenario model” and “scenarios” in Figure 1). This scenario grammar can generate scenarios with more maneuverability compared with existing straight line or Markov motion models [7,13]. In terms of Challenge (2), this scenario grammar can generate a low-dimensional form of the traditional testing cases in existing simulation or AIS-based methods, represented in a concise sequence of parameterised events (also known as a scenario depicting the maritime situation) instead of the high-fidelity sampled states of ship targets (also known as the simulations or the transformed AIS data).

Challenge (3) is tackled by introducing a closed-loop feedback using evolutionary computation (EC) to manipulate the scenario model to generate new scenarios that can cause more and more tracking performance loss (see “evolutionary computation” in Figure 1). Various cases or datasets can be collected during this evolutionary process for further analysis. **We believe that the collected scenarios, which have caused serious tracking performance loss, can provide us with valuable testing data in terms of multiple maneuvering ship targets. This can also provide insights into possible vulnerability in our tested ship tracking algorithm wherein focus can be put on to improve tracking performance.**

Our preliminary work [9] has demonstrated the effectiveness of our proposed evolutionary approach in terms of the above three challenges. In this paper, we improve this approach by taking the HFSWR capabilities and the uncertainties in the ship detection process into account, analysing the scenario dataset and investigating the impact of motions of multiple ship targets on the tracking performance by comparing different maneuver models [14] and tracking algorithms. We analyse the collected scenario datasets with different degrees of TF using trajectory mining technique and, finally, discover quantitative metrics in our proposed three motion patterns of ship targets as possible indicators of the vulnerability in the tested HFSWR ship-tracking algorithm (see “datasets” and “vulnerability descriptions” in Figure 1). **The discovered indicators of tracking vulnerability have the potential to suggest future directions towards where to make efforts to improve ship-tracking performance and trigger early-warning control in real-time maritime surveillance.**

We need to clarify that the focus of this paper is to investigate the performance of the ship tracking process, especially the effects of complex ship motions on tracking. However, the HFSWR capabilities and the uncertainties in the ship detection process still need to be taken into account in order to reflect the practical HFSWR ship detection and tracking environment. This is achieved in our scenario generation process as follows: first, the values of ships’ kinematic information are set according to the matching AIS data with practical HFSWR ship detection results; second, the position of each ship in a scenario is set by cells to conform to the range and azimuth resolution of the HFSWR system; and third, background noise and false ship targets are further added to the simulated ship motion states.

The rest of the paper is organised as follows. Section 2 briefly addresses Challenges (1) and (2), in which we generate practical scenarios of any number of ships moving autonomously in a certain sea area as the cases for testing the performance of a HFSWR ship-tracking algorithm. These generated scenarios are then evolved using a specific type of EC to collect scenario datasets with different degrees of TF in Section 3 to tackle Challenge (3). Section 4 presents the definitions of our proposed three motion patterns of ships—direction-confined convoy, snapshot cluster and convergence. Details of the experimental design and result analysis are presented in Section 5, in which quantitative performance evaluation and vulnerability detection of HFSWR ship-tracking algorithms are shown. Results and future work are discussed in Section 6. Finally, some concluding remarks are made in Section 7.

## 2. Scenario Modelling and Generation

The objective of this section is to generate various scenarios that depict multiple ships moving autonomously in the surveillance sea area, also known as the cases or datasets for testing the performance of a HFSWR ship-tracking algorithm. Challenge (1) of existing performance assessment works on HFSWR ship-tracking is addressed by proposing a scenario model based on context-free grammar (CFG) [15], which has been elaborated in our previous work [9].

Compared with existing motion models in the simulation-based assessment approach, our scenario model can generate lower-dimensional scenarios because this model captures the motions of ships on the semantic level. First, the building blocks of this model are concise, which are composed of the initial states and a sequence of events (i.e., changes of states) that could happen in the motions of each ship. Second, those building blocks of scenarios are combined by a set of grammatical rules, which indicate the relationships between them to form a coherent whole.

An example of the generated scenarios skeleton is “$t_{0}\;i_{0}\left(1\right)\;t_{\Delta }\left(1\right)\;d_{\Delta }\left(1\right)\;\varepsilon \;t_{d}\left(1\right)\;\left[\;i_{0}\left(2\right)\;t_{\Delta }\left(2\right)\;v_{\Delta }\left(2\right)\;\varepsilon \;t_{d}\left(2\right)\;\left[\;i_{0}\left(3\right)\;t_{\Delta }\left(3\right)\;d_{\Delta }\left(3\right)\;\varepsilon \;\right]\;\right]$”. The scenario skeleton can be explained as: Ship 1 appeared at t=$t_{0}$ in initial state $i_{0}\left(1\right)$, changed its direction by $d_{\Delta }\left(1\right)$ degree after $t_{\Delta }\left(1\right)$ time period, and kept moving without any change of direction or speed. After $t_{d}\left(1\right)$ since Ship 1 firstly appeared, Ship 2 showed up in initial state $i_{0}\left(2\right)$, changed its velocity by $v_{\Delta }\left(2\right)$ km/h, and kept moving. After $t_{d}\left(2\right)$ since Ship 2 firstly appeared, Ship 3 started to play in initial state $i_{0}\left(3\right)$, changed its direction by $d_{\Delta }\left(3\right)$ degree, and kept moving.

A scenario is obtained by instantiating the building blocks of the generated scenario skeleton with values of ships’ kinematic information extracted from the matching AIS data with practical HFSWR ship detection results to ensure that they are in accord with real ship motion characteristics (see Section 5.1 for the value ranges).

## 3. Scenario Evolution

This section addresses Challenge (3) of existing performance assessment works on HFSWR ship-tracking by designing an appropriate evolutionary computation (EC) algorithm to introduce a closed-loop feedback into the scenario generation process, so that new generations of scenarios that can cause more and more tracking performance loss can be collected as cases for testing the performance of a HFSWR ship-tracking algorithm.

### 3.1. EC Design for Scenario Evolution

Evolutionary computation (EC) [16] is a type of intelligent search and optimisation technique, which imitates the biological evolution in nature. The design of any EC algorithm usually involves the “encoding and decoding scheme”, which represents any testing case—the scenario—in a way that evolution can operate on; the “genetic operators”, which include a selection method to select one or more already-generated scenarios (also known as the individuals) from the pool (the population), and the operators that can make changes to the selected scenarios to generate new ones; and the “fitness” function, which evaluates each generated scenario to provide feedback information for the EC to manipulate the scenario model towards generating the scenarios of our interest.

The grammar representation of our scenario model facilitates the design of a particular type of EC—grammar-guided genetic programming (GGGP) algorithm. A revised GGGP algorithm has been designed and presented in our previous work [9].

### 3.2. Scenario Evaluation with Added Background Noise and False Targets

We improve our evolutionary approach by taking the background noise and false targets into consideration in this paper. A revised scenario evaluation process is proposed as follows, as illustrated in Figure 1.

The scenarios are firstly transformed into simulated ship motions through the decoding process explained in Ref. [9].

Background noise and false targets are added to the simulation data, which are compressed into the input of the ship tracking algorithm. The algorithm outputs the tracking results and tracking performance metrics, such as number of track fragmentation ($n_{TF}$), can be calculated. It is worth mentioning that existing ship tracking performance metrics need to be transformed considering that the objective of our evolution is to detect tracking vulnerability—to find scenarios that can cause performance loss in the ship tracking algorithm.

The fitness function $f_{1}$ is defined in Equation (1), which measures the average number of track fragmentation (TF) [7,8,10] caused by a scenario. It is defined based on the fact that, if the ship-tracking algorithm works perfectly, the output number of track fragments for each ship will be 1, without any subtracks. The bigger the value of $f_{1}$ is, the worse the tracking performance is. *N* denotes the number of ships in the scenario and $n_{TFi}$ is the number of track fragments of a ship labeled by *i* calculated from the output of the HFSWR ship-tracking algorithm.

(1) $$f_{1}=\frac{1}{N}\sum_{i=1}^{N}n_{TFi}$$

## 4. Motion Patterns and Trajectory Mining

Scenarios that can cause different degrees of track fragmentation (TF), especially those that can cause severe TF failures, are collected from the evolution process proposed in the last section. The objective now is to investigate potential quantitative indicators of tracking failure such as TF. The collected scenarios during the evolutionary process can provide us with the testing cases or datasets of the HFSWR tracking algorithm, so that we can perform further analysis to achieve this.

Three motion patterns—direction-confined convoy, snapshot cluster and convergence—inspired by existing motion patterns in trajectory mining domain [17,18] are proposed in this section. We conjecture that some metrics of these motion patterns may serve as potential quantitative indicators of the TF tracking failures.

### 4.1. Motion Patterns and Density-Based Clustering

**Figure 2:**
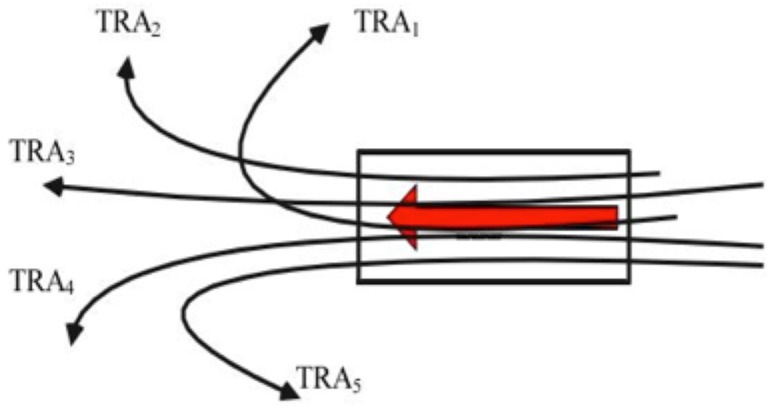
Example of motion pattern.

Motion patterns denote the motion characteristics of common sub-trajectories extracted from a trajectory cluster of moving point objects [17], which is the product of trajectory pattern mining [18]. As shown in Figure 2, trajectories TR1–TR5 show different movement situations while sharing similar sub-trajectories or “motion patterns” in the box.

**Figure 3:**
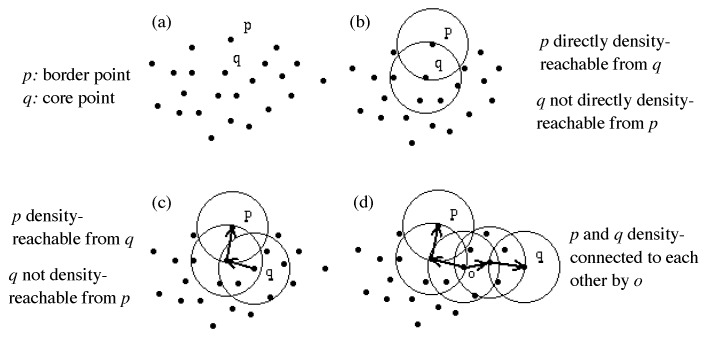
Directly density-reachable, density reachable and density-connected.

Now, we adopt the notion of density-based clustering in the DBSCAN algorithm [19] to define the “convoy” and “snapshot cluster” patterns. The key idea of density-based clustering is that for each point of a cluster, the neighbourhood of a given radius Eps has to contain at least a minimum number of points, i.e., the density in the neighbourhood has to exceed some threshold MinPts. Following are the formalised definitions [19] related to the notion called “density-connected”, which is fundamental to our proposed “convoy” and “snapshot cluster” patterns (see Figure 3 for illustration).

**Definition** **1:** (Eps-neighbourhood of a point *p*) denoted by $N_{Eps}\left(p\right)$, is defined by $N_{Eps}\left(p\right)=\left\{q\in D|dist\left(p,q\right)\leq Eps\right\}$, in which *D* denotes a database of points of some k-dimensional space including *p* and *q*, and dist(p,q) is a distance function for two points *p* and *q*.

**Definition** **2:** (directly density-reachable) A point *p* is directly density-reachable from a point *q* with respect to Eps, MinPts if

1. $p\in N_{Eps}\left(q\right)$ and
2. $\left|N_{Eps}\left(q\right)\right|\geq MinPts$ (core point condition).

**Definition** **3:** (density-reachable) A point *p* is density-reachable from a point *q* with respect to Eps and MinPts if there is a chain of points $p_{1},\ldots ,p_{n}$, $p_{1}=q$, $p_{n}=p$ such that $p_{i+1}$ is directly density-reachable from $p_{i}$.

**Definition** **4:** (density-connected) A point *p* is density-connected from a point *q* with respect to Eps and MinPts if there is a point *o* such that both *p* and *q* are density-reachable from *o* with respect to Eps and MinPts.

### 4.2. Direction-confined Convoy Pattern

We propose the “direction-confined convoy” pattern to represent the motion characteristics of sub-trajectories when close objects move in approximate parallel in a continuous period of time. It is defined as at least two objects moving while maintaining their locations density-connected (the MinPts and Eps threshold of DBSCAN is set to 2 and 10 km, respectively) and their direction within a range (30${}^{\circ }$) in a continuous period of time (5 min). It is worth noting that the above thresholds can be modified according to different concerns.

This “direction-confined convoy” pattern (which is referred to as “convoy” pattern in the rest part of this paper) is revised from an existing convoy pattern proposed by Jeung et al. [20] by further confining the moving directions of the objects in the sub-trajectories to a range.

### 4.3. Snapshot Cluster Pattern

We use the existing “snapshot cluster” pattern proposed by Zheng et al. [21] to represent the motion characteristics of sub-trajectories when objects gather together at a given timestamp. It is defined as at least three objects density-connected to each other at a given timestamp (the MinPts and Eps threshold of DBSCAN is set to 3 and 10 km, respectively).

### 4.4. Snapshot Convergence Pattern

We propose the “snapshot convergence” pattern to represent the motion characteristics of close objects moving in an intersecting way at a given timestamp. It is defined as a group of objects (at least two) move with motion azimuth vectors intersecting within a range of radius *r* (1 km) at a given timestamp.

This snapshot convergence pattern is revised from existing convergence pattern proposed by Laube et al. [17], which allows convergence within a time period rather than at a timestamp.

## 5. Results

We present the results we obtained to show the effectiveness of our proposed evolutionary and data-mining-based approach in collecting high-fidelity testing datasets (also known as scenario datasets) with different degrees of tracking performance characteristics, achieving intensive and quantitative performance assessment of a HFSWR ship tracking algorithm, and in discovering indicators of tracking vulnerability by analysing these datasets. It is worth noting that results of different maneuver models and tracking algorithms, with and without added noise, are presented and compared to verify our findings.

### 5.1. Experimental Design

Performance assessment and vulnerability detection was implemented on two types of tracking algorithms—the converted measurement Kalman filter [22,23,24] and the Gaussian mixture probability hypothesis density filter (GM-PHD) [25,26]. In the former Kalman filter-based tracking algorithm, results of different maneuver models [14] were compared, which include the classical CV and CA models, and the Singer model. As the original GM-PHD-based tracking algorithm does not provide identities of individual target state estimates, which are needed to construct tracks of individual targets, we further associated among state estimates of targets over time and provide track labels [27]. Results of four tracking algorithms—CV model, CA model and Singer model based on converted measurement Kalman filter, and GM-PHD—are provided and compared.

**Figure 4:**
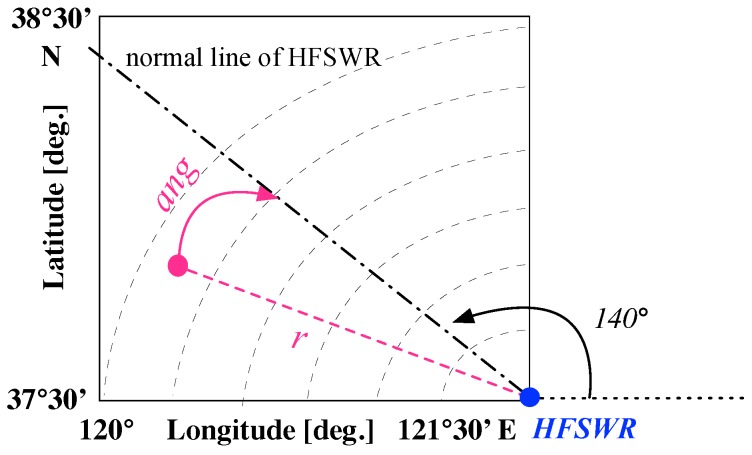
Coordinate system setting.

The coordinate system setting is illustrated in Figure 4. The HFSW radar is located in the blue point at the bottom right of the geographic coordinate system, with its normal line $140^{\circ }$ to the longitude axis of the geographic coordinate system. The discussed surveillance area is denoted by the square.

The scenarios are generated based on the following value ranges extracted from the matching AIS data with practical HFSWR ship detection results. The practical dataset was collected from the “Weihai” site in China. The site operates at 4.7 MHz with a bandwidth of 100 kHz. The range resolution is 1.5 km. The dataset is composed of measurements obtained for five days. It can be observed that the ships are constrained to comparatively small maneuverability, which can be easily modified to allow more maneuverability and probably cause severer TF. Moreover, to generate scenarios that conform to the real HFSWR capacities in terms of range and azimuth resolution in the detection stage, the surveillance area was divided into 60 (in range) × 9 (in azimuth) cells. The initial position of each ship in a scenario was set by cells accordingly.

- Initial velocity: 5∼35 km/h
- Initial positions: longitude 120${}^{\circ }$ E∼121${}^{\circ }$30${}^{\prime }$ E, latitude 37${}^{\circ }$30${}^{\prime }$ N∼38${}^{\circ }$30${}^{\prime }$ N
- Velocity change: −1.55 km/h∼2.25 km/h
- Direction change: −10${}^{\circ }$∼10${}^{\circ }$
- Ship number limit: 50
- Simulation time range: 4 h
- Simulation sampling time: 1/60 h

The background noise was defined by a zero-mean Gaussian process [28]. As the correlation wave gate threshold of the tested HFSWR ship tracking algorithm was set to 2 km for the range *r*, 1 km/h for the radial velocity $v_{r}$, and 3.2${}^{\circ }$ for the azimuth relative to boresight ang, the following background noise was added to the motion state points of the ship targets at every sampling time to avoid exceeding these thresholds.

- b(r,t)∼N[0,0.3] km
- $b\left(v_{r},t\right)∼N\left[0,0.05\right]$ km/h
- $b(ang,t)∼N[0,0.5]{}^{\circ }$

Fitness function in terms of track fragmentation defined in Equation (1) was applied to guide the scenario evolution. After testing the performance of the GGGP under different parameter settings, the following were selected. As the scenario representation of our work was comparatively complex, incorporating the initial motion states and events of multiple ship targets, the mutation rate was set to be high. One reason is that the mutation operator plays an important role in maintaining the diversity of the scenario population. Moreover, our designed mutation operators can make small changes to the generated scenario by, for instance, changing the initial velocity value of a ship target or deleting a direction change from its motions.

- Population size: 20
- Generation limit: 400
- Generation gap: 0.5
- Crossover rate: 0.7
- Mutation rate: 0.3, in which the node replacement and value change of the terminal were both implemented

Two types of simulation data of the collected scenario datasets were analysed: simulation data with and without added noise. We need to clarify that the simulation data used for trajectory mining were the ground truth data, i.e., the motion state points of the ship targets at every sampling time (also known as the “trajectory” data in the trajectory mining domain) rather than the input of the tracking algorithm.

The scenario datasets were collected using the evolutionary method proposed in Section 3 and sampled in the following way: we firstly ran the GGGP guided by fitness function $f_{1}$ defined in Equation (1) 40 times using 40 different seeds. We then chose the best 20 runs as our data pool, in which the fitness value of the best individual in the final generation of each run was higher than those in the other runs. There were maximum 80,400 scenarios in total (“20 individuals/scenarios in the initial population + 20 individuals × 0.5 generation gap × 400 generations” in each run). We further ranked the scenarios in the initial population (i.e., the randomly generated scenarios, also known as Generation 0) of all 20 runs according to their fitness values, which added up to 400 scenarios, as well as those in the final population (i.e., Generation 400). Subsequently, we uniformly chose 50 scenarios from the ranked scenarios at the same interval in the initial and the final population, respectively, and obtained two sampling datasets: the “initial” and “final” sampling datasets.

All scenarios and their simulated data obtained during evolution could be analysed, which shows one advantage of our evolutionary approach in collecting high-fidelity testing datasets for the HFSWR ship-tracking algorithm. In this study, we focused only on the randomly generated scenarios and the fully evolved scenarios—scenarios that can cause severe TF failures to the ship-tracking algorithm—and compared them to find quantitative indicators of the TF tracking failures.

**Figure 5:**
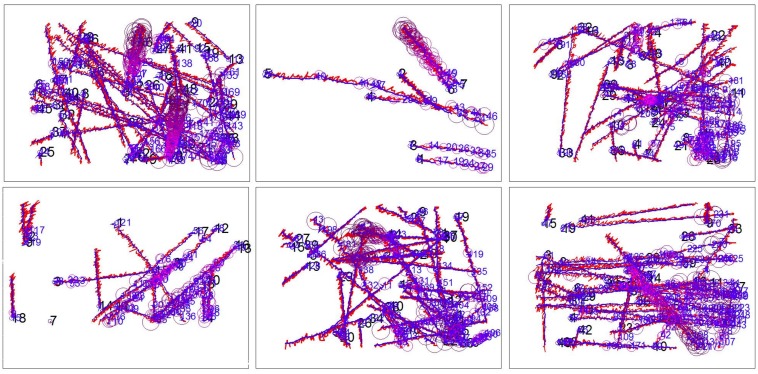
Example scenarios in the “final” sampling dataset (with noise): the red lines denote the true trajectories of ships with added noise, the blue lines denote the output tracks of the HFSWR ship-tracking algorithm, and the circles mark the fragmentation points of the tracks, with the bigger size and darker colour denoting later sampling time.

Figure 5 illustrates six example scenarios in the 50 “final” sampling dataset (with noise), which indicates diversity in the evolved scenarios or scenarios that can cause severe TF failures. As the original trajectory and track plot can only show the spatial information, we introduce circles of different sizes and colours to indicate the temporal information of the fragmentation points in the tracks—bigger size and darker colour denote later sampling time. In this way, we could incorporate spatial and temporal information in one trajectory and track plot in HFSWR ship-tracking domain.

False targets in different proportions were added to the simulated data (with noise) of the sampling dataset and their effects on the tracking performance were tested and compared.

### 5.2. Failure-Scenario Collection Experiments

Experiments were conducted to verify our proposed evolutionary method in its ability to automatically collect scenario datasets that can cause different degrees of TF failures to a HFSWR ship-tracking algorithm, especially scenarios that can cause severe TF failures and reveal vulnerability of the tracking algorithm. The performance of our designed GGGP in Section 3 was tested and this evolutionary method was compared with Monte Carlo simulation method.

It is worth noting that the above-mentioned four tracking algorithms—CV model, CA model and Singer model based on converted measurement Kalman filter, and GM-PHD—share similar fitness transitions, while exhibiting different fitness value ranges in the final population, which shows difference in the tracking performance.

**Figure 6:**
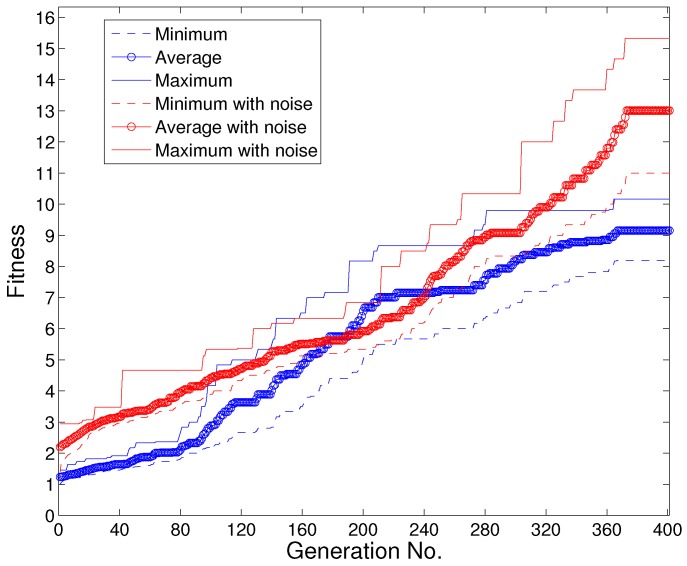
Transition of track fragmentation fitness $f_{1}$ (with vs. without noise).

The transitions of the fitness function $f_{1}$ defined in Equation (1) during the evolutionary process of a typical run of the ship tracking algorithm based on Kalman filter and CV model is illustrated in Figure 6. It includes plots of the minimum, average and maximum values of the fitness function among the scenarios in the population of each generation as evolution proceeds. The red lines denote the TF fitness transitions with added noise to the simulation data of the scenarios, while the blue lines denote those without noise.

It can be observed that the evolutionary process succeeded in evolving failure-scenarios, i.e., collecting scenarios with severer track fragmentation, reflected in the increasing trend in the plots in this figure. In addition, the diversity of the scenarios in each generation was maintained considering the wide distribution of the fitness values in each generation. Moreover, evolutionary process with added noise to the simulation data of the scenarios could discover scenarios that can cause severer TF failures to the ship-tracking algorithm, reflected in the much higher fitness values of the red plots compared with the blue plots in the final generation (Generation No. = 400), which is reasonable considering that noise can interfere with the tracking initiation, correlation and filtering stage.

**Figure 7:**
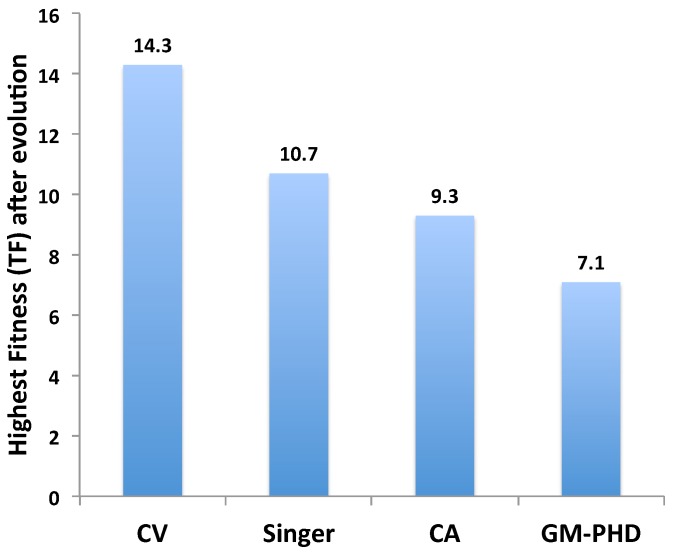
Highest fitness (TF degrees) after evolution of four tracking algorithms (with noise).

Comparison of the four tracking algorithms in terms of the highest fitness values (TF degrees) after evolution is given in Figure 7. It shows that GM-PHD tracking algorithm enjoyed the lowest TF degrees under the above settings.

**Figure 8:**
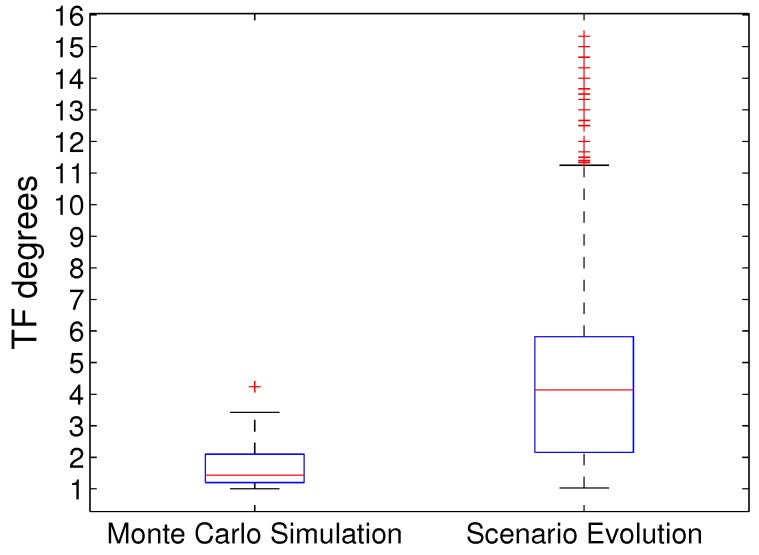
Distributions of TF degrees in scenario datasets collected by Monte Carlo simulation method and our evolutionary method.

The scenario datasets collected by the Monte Carlo simulation method in existing ship tracking works and our evolutionary method are compared in Figure 8 in terms of the distributions of TF degrees. The two datasets were obtained using the same scenario settings in Section 5.1. The Monte Carlo simulation process were conducted 3956 times to collect 3956 diversified scenarios, while our evolutionary process was run once to collect the unique 3956 scenarios in all generations (maximum 4020 scenarios = 20 in the initial population + 20 × 0.5 generation gap × 400 generations). Our evolutionary method significantly outperformed the Monte Carlo simulation method in collecting scenario dataset with dramatically higher and wider distributions of TF degrees.

**Figure 9:**
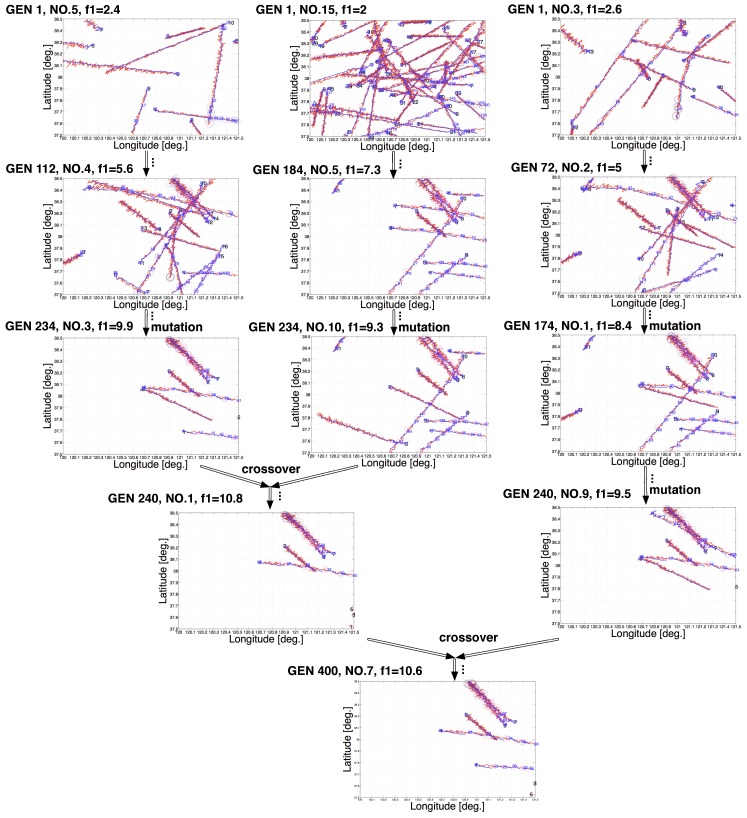
Partial family tree of an evolved scenario (with noise): the directed lines point from one scenario to its descendant(s) generated from genetic operations such as crossover and mutation as the evolutionary process proceeds; the labels on the directed lines denote the specific genetic operations implemented to produce the descendant(s), in which “...” denotes ellipsis in the full family tree due to the space limit; GEN denotes the generation label of the evolutionary process of the scenario, where the bigger is the GEN, the more evolved is the scenario; No. denotes the individual label of the scenario in the population; and $f_{1}$ shows the fitness value of this scenario.

As the evolutionary process of scenarios discussed in Section 3 proceeded, new generations of scenarios were produced by mutating one scenario and/or crossing over two scenarios in the last generation, in which a family tree could be drawn. Figure 9 illustrates a partial family tree of an evolved scenario (of the CV model based on converted measurement Kalman filter) located at the bottom of the figure, in which the same annotations to Figure 5 are applied. It shows that in the initial population (see scenarios on the top of the family tree), tracks are seldom broken, reflected in the few circles (i.e., the fragmentation points of the tracks) and smaller $f_{1}$ values, which indicates good overall tracking performance, while later descendants inherit but differ from their ancestors in the trajectory form and exhibit gradually severer TF failures as $f_{1}$ values increase.

### 5.3. False Targets Experiments

Detection of ship targets using HFSWR systems may suffer from significant false alarm rate due to both sea clutter and man-made/natural interference, which may have indirect impact on the performance of the ship-tracking algorithm by manipulating its inputs. Experiments were carried out to investigate the effects of false targets on ship tracking performance.

False targets in different proportions (10%, 20% and 30%) were added, respectively, to the simulated data (with noise) in the above-mentioned sampling dataset of the GM-PHD tracking algorithm, which enjoyed the lowest TF degrees. At each sampling time, the range, azimuth and radial velocity of the false targets were produced in uniform distribution and added to the real simulated data. The tracking performance based on the three dataset were then tested and compared.

**Figure 10:**
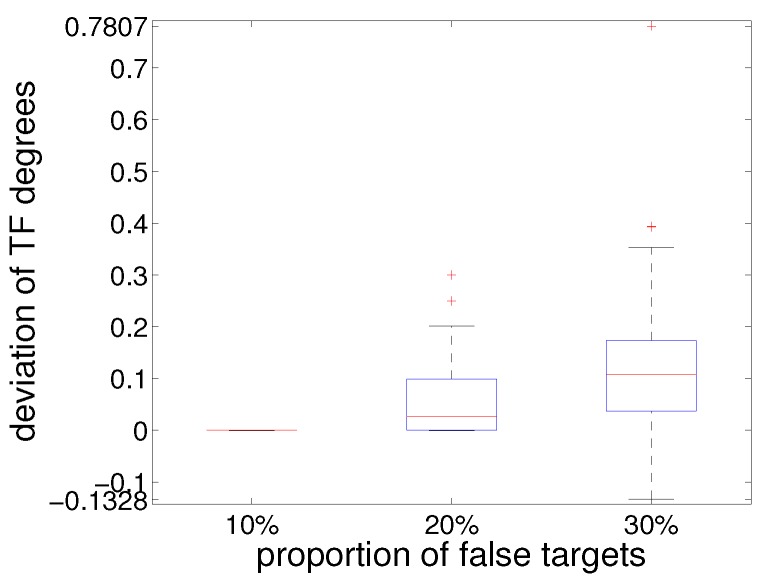
Distributions of deviation of TF degrees in sampling dataset with false targets from that without false targets.

Figure 10 presents the distributions of deviation of TF degrees in the above sampling dataset with false targets from that without false targets. It shows that, in general, TF degrees increased when false targets were introduced, with only a minority of data exhibiting negative deviation of TF degrees. Ten per cent false targets had negligible effect on the tracking performance in terms of TF degrees. As the proportion of false targets increased from 20% to 30%, a higher and wider distributions of TF deviations was noticed, with the maximum deviation increasing from 0.3000 to 0.7807.

### 5.4. Trajectory Mining Experiments

The collected scenario datasets were further analysed using trajectory mining technique discussed in Section 4, in which our proposed three motion patterns of ship targets were identified with their quantitative metrics calculated and their impact on HFSWR tracking performance discussed.

**Figure 11:**
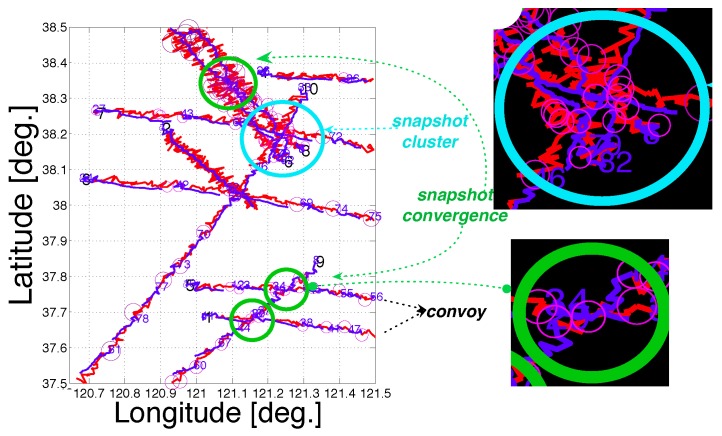
Three motion patterns in scenario (with noise) “GEN 174, No. 1, $f_{1}$ = 8.4”.

The three motion patterns could be frequently observed from the collected scenarios through evolution. Figure 11 illustrates the convoy, snapshot cluster and snapshot convergence patterns in scenario “GEN 174, No. 1, $f_{1}$ = 8.4” in the partial family tree in Figure 9. In the enlarged details in the top right of this figure, we can observe a snapshot cluster pattern in which there exist concentrated TF points of four different ships at the same time, reflected from the same circle size, which indicates TF at the same timestamp. The bottom right of this figure shows a typical TF failure caused by failures in the internal correlation process of tracking. A snapshot convergence pattern can be identified, in which the trajectories of two ships intersected with each other at a timestamp, so that the tracking algorithm broke their tracks into fragments and, then, misconnected them to each other by mistake. This example indicates that TF can also be caused by motion patterns such as convoy, snapshot cluster and convergence patterns except for the lack of target contacts for some periods of time.

Table 1 compares the statistics of the snapshot cluster pattern before (Generation 0) and after evolution (Generation 400) for the four tracking algorithms, while Table 2 compares that of the snapshot convergence pattern. Both tables show that the evolved scenarios contained more snapshot cluster and convergence patterns than the randomly generated scenarios, reflected from the larger percentage of scenarios with patterns and the larger number of patterns in the datasets. The statistics implies that the number of snapshot cluster and convergence patterns in the scenarios may be related to the degree of track fragmentation failures to some degree, which is not obvious for the convoy pattern.

**Figure 12:**
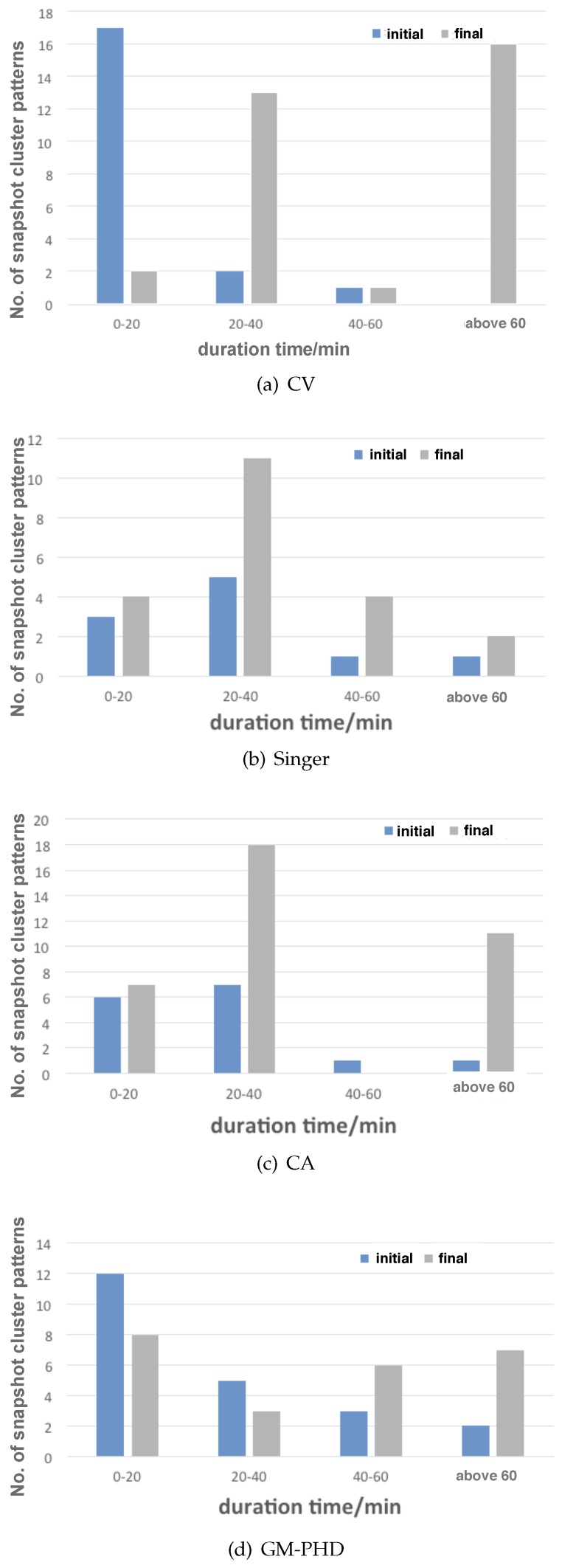
Distributions of duration time for the snapshot cluster patterns before and after evolution (with noise).

**Figure 13:**
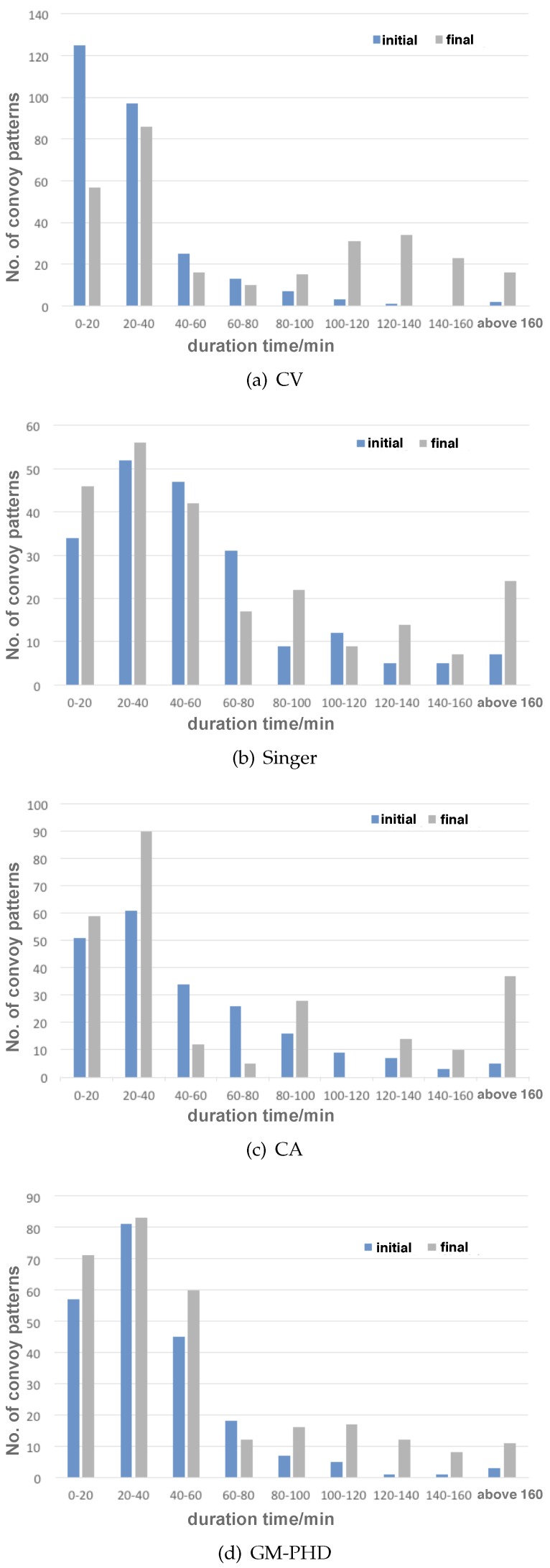
Distributions of duration time for the convoy patterns before and after evolution (with noise).

It is interesting to notice that the duration time of the snapshot cluster patterns in the evolved scenarios in the final generation was exclusively long compared with that of the randomly generated scenarios in the initial generation (see Figure 12). Similarly, direction-confined convoy patterns that last for more than 100 min were seldom observed in the initial population compared with considerable distributions of long-time convoy patterns in the final population (see Figure 13). The above results indicate that longer duration time of the snapshot cluster patterns and the direction-confined convoy patterns could cause severer track fragmentation failures to the HFSWR ship-tracking algorithm, which could probably be traced back to failures in the internal correlation stage of tracking and requires further research.

We also investigated the distributions of the moving directions and the difference in the moving directions between two ships, the ranges, the azimuths relative to boresight and the difference in the azimuths relative to boresight between two ships to exclude their effects on the HFSWR ship-tracking algorithm. Results show no noticeable distribution patterns between scenarios before and after evolution.

## 6. Discussion

Results of four tracking algorithms—CV model, CA model and Singer model based on converted measurement Kalman filter, and GM-PHD—show that our evolutionary method significantly outperformed existing simulation method of ship-tracking performance assessment in collecting a scenario dataset with dramatically higher and wider distributions of track fragmentation (TF) degrees—a typical ship-tracking performance loss.

The above four tracking algorithms shared similar upward fitness transitions during the evolutionary process, which verified our evolutionary approach in evolving failure-scenarios, i.e., collecting scenarios with severer track fragmentation. Noise in the kinematic information of ships could cause severer TF failures to the HFSWR ship-tracking algorithm.

In addition, the four tracking algorithms exhibited different fitness value ranges in the evolved final population, which indicates difference in the tracking performance. GM-PHD tracking algorithm enjoyed the lowest TF degrees under the above experimental settings, followed by the CA model, Singer model and CV model based on converted measurement Kalman filter.

In general, TF degrees increased when false targets were introduced. As the proportion of false targets increased, a higher and wider distributions of TF deviations could be noticed.

Moreover, we found from the trajectory mining results of all four tracking algorithms that TF could also be caused by motion patterns that represent close objects moving in approximate parallel in a continuous time period (the direction-confined convoy pattern), objects gathering together (the snapshot cluster pattern) or moving in an intersecting way (the snapshot convergence) at a timestamp. The number of our proposed snapshot cluster and convergence patterns in the scenarios may be related to the degree of TF tracking failures to some degree; in addition, longer duration time of the snapshot cluster patterns and the convoy patterns could cause severer TF failures, which could probably be traced back to failures in the internal correlation stage of tracking.

At this stage, the way we represent the HFSWR limitations and the uncertainties in the ship detection process to investigate their indirect impact on ship tracking may be inappropriate. For instance, only minor background noise and random motion states of the false targets have been added to the input of the tracking algorithm. Nowadays, it is still a non-trivial task to simulate a practical HFSWR ship detection and the tracking environment thus requires further research. Future work includes setting more practical noise, loss targets and false targets according to different HFSWR settings and environment. For example, the ionospheric variation exhibits centralised distributions of range and wide distributions of radial velocities; and the accuracy of the detected kinematic information of ship targets is significantly affected by the signal to clutter ratio (SCR), which is related to the range of the ship target, the range and azimuth resolution of the HFSWR system, as well as the sea state. Moreover, only one HFSWR setting has been considered in this paper in terms of frequency, bandwidth and number of received elements, which the range and azimuth resolution is dependent on. Future work involves generating ship motion scenarios according to diversified range and azimuth resolutions based on different HFSWR settings. This can be achieved in our scenario-to-simulation transformation process, by classifying the ranges and azimuths of ships precisely calculated from the scenario on each sampling time into corresponding range and azimuth cells as the simulation results. What is more, the impact of other HFSWR limitations and capabilities in relation to the radar sampling strategy, ship orientation, radar geometry, etc. on the ship tracking performance needs to be investigated in detail, probably by going deep into the signal level.

As we regard the HFSWR ship-tracking algorithm for performance assessment as a black box in this paper, vulnerability detection is only based on the external ground-truth scenario and simulation data instead of going into the internal tracking operations such as the tracking initiation, correlation and filtering stages. Our approach provides an effective platform and facilitates future research on testing the performance and detecting the vulnerability of a HFSWR ship-tracking algorithm by interacting with its internal operations.

Future work involves defining shipping lanes in the scenario generation process, which can be realised by confining the initial states of the ship targets in the scenario grammar, such as their initial positions, moving directions, etc., and by redefining the surveillance area. Future work also includes introducing more trajectory shapes (e.g., arcs, not restricted to combinations of straight lines in this paper) or motion models to the grammar to enhance the scenario model. Other tracking characteristics except for TF can also be investigated by simply changing the fitness function.

## 7. Conclusions

Motions of ship targets can significantly affect the performance of a HFSWR ship-tracking algorithm. However, there is a lack of research on understanding when, how and to what degree tracking failures can occur. This paper addresses this problem and attempts to undertake a first step towards providing an intensive quantitative performance assessment and vulnerability detection scheme for ship-tracking algorithms by proposing an evolutionary and data-mining-based approach. This approach is promising in collecting diversified testing cases in terms of multiple maneuvering ship targets for ship-tracking algorithms and discovering tracking vulnerabilities, wherein we can make efforts to improve ship-tracking performance and trigger early-warning control in real-time maritime surveillance.

To solve the problems in existing simulation or AIS-based performance assessment methods, we firstly generated practical and low-dimensional scenarios of any number of ships moving autonomously in a certain sea area as the cases for testing the performance of a HFSWR ship-tracking algorithm, in which the HFSWR capabilities and the uncertainties in the ship detection process are taken into account. We then introduced a closed-loop feedback using evolutionary computation (EC) to search for cases with varying degrees of performance characteristics, especially those scenarios that could cause severe tracking performance loss. The collected scenarios provide us with valuable testing data, which we analysed using trajectory mining technique, with quantitative indicators of tracking vulnerability discovered.

Results of four different maneuver models and tracking algorithms verify our findings and proposed performance evaluation scheme for multiple object tracking.

## Figures and Tables

**Table 1:** Statistics of snapshot cluster pattern (with noise) for the four tracking algorithms.

Algorithm	Generation	$f_{1}$ Fitness Value	Data Size	Data with Pattern (%)	No. of Patterns
CV	0 (initial)	1.5∼2.9	50	26%	38
CV	400 (final)	5.3∼14.3	50	32%	64
Singer	0 (initial)	1.5∼3.3	50	20%	14
Singer	400 (final)	5.8∼10.7	50	36%	21
CA	0 (initial)	1∼3.3	50	18%	15
CA	400 (final)	4.6∼9.3	50	46%	36
GM-PHD	0 (initial)	1∼3.2	50	20%	16
GM-PHD	400 (final)	3.6∼7.1	50	30%	22

**Table 2:** Statistics of snapshot convergence pattern (with noise) for the four tracking algorithms.

Algorithm	Generation	$f_{1}$ Fitness Value	Data Size	Data with Pattern (%)	No. of Patterns
CV	0 (initial)	1.5∼2.9	50	64%	158
CV	400 (final)	5.3∼14.3	50	82%	194
Singer	0 (initial)	1.5∼3.3	50	68%	124
Singer	400 (final)	5.8∼10.7	50	70%	166
CA	0 (initial)	1∼3.3	50	62%	118
CA	400 (final)	4.6∼9.3	50	72%	205
GM-PHD	0 (initial)	1∼3.2	50	70%	134
GM-PHD	400 (final)	3.6∼7.1	50	74%	157

## Abbreviations

The following abbreviations are used in this manuscript:

HFSWR	High-Frequency Surface Wave Radar
FAR	False Alarm Rate
CFAR	Constant False Alarm Rate
DOA	Direction Of Arrival
MTT	Multitarget Tracking
TF	Track Fragmentation
AIS	Automatic Identification System
CFG	Context Free Grammar
EC	Evolutionary Computation
GGGP	Grammar-Guided Genetic Programming
GM-PHD	Gaussian Mixture Probability Hypothesis Density filter
SCR	Signal to Clutter Ratio
